# Sustainable Electropolymerization of Zingerone and Its C2 Symmetric Dimer for Amperometric Biosensor Films

**DOI:** 10.3390/molecules28166017

**Published:** 2023-08-11

**Authors:** Myriam Caval, Maria Antonietta Dettori, Paola Carta, Roberto Dallocchio, Alessandro Dessì, Salvatore Marceddu, Pier Andrea Serra, Davide Fabbri, Gaia Rocchitta

**Affiliations:** 1 Dipartimento di Scienze Biomediche, Università Degli Studi di Sassari, 07100 Sassari, Italy; m.caval@studenti.uniss.it; 2 Istituto di Chimica Biomolecolare, Consiglio Nazionale Ricerche, 07100 Sassari, Italy; mariaantonietta.dettori@cnr.it (M.A.D.); paola.carta@cnr.it (P.C.); robertonico.dallocchio@cnr.it (R.D.); alessandro.dessi@cnr.it (A.D.); 3 Istituto di Istituto Scienze delle Produzioni Alimentari, Consiglio Nazionale Ricerche, 07100 Sassari, Italy; salvatore.marceddu@ispa.cnr.it; 4 Dipartimento di Medicina, Chirurgia e Farmacia, Università Degli Studi di Sassari, 07100 Sassari, Italy; paserra@uniss.it

**Keywords:** biosensor, zingerone, zingerone dimer, electropolymerization, molecular docking

## Abstract

Polymeric permselective films are frequently used for amperometric biosensors to prevent electroactive interference present in the target matrix. Phenylenediamines are the most commonly used for the deposition of shielding polymeric films against interfering species; however, even phenolic monomers have been utilized in the creation of these films for microsensors and biosensors. The purpose of this paper is to evaluate the performances of electrosynthesized polymers, layered by means of constant potential amperometry (CPA), of naturally occurring compound zingerone (ZING) and its dimer dehydrozingerone (ZING DIM), which was obtained by straight oxidative coupling reaction. The polymers showed interesting shielding characteristics against the main interfering species, such as ascorbic acid (AA): actually, polyZING exhibited an AA shielding aptitude comprised between 77.6 and 99.6%, comparable to that obtained with PPD. Moreover, a marked capability of increased monitoring of hydrogen peroxide (HP), when data were compared with bare metal results, was observed. In particular, polyZING showed increases ranging between 55.6 and 85.6%. In the present work, the molecular structures of the obtained polymers have been theorized and docking analyses were performed to understand their peculiar characteristics better. The structures were docked using the Lamarckian genetic algorithm (LGA). Glutamate biosensors based on those polymers were built, and their performances were compared with biosensors based on PPD, which is the most widespread polymer for the construction of amperometric biosensors.

## 1. Introduction

Precisely measuring the analyte concentration requires methods that are selective, fast, and robust. This is crucial for process control, product analysis, environmental compliance, and medical applications. Enzyme-based biosensors are well suited for these purposes, as they provide high accuracy and can be operated with simple, cost-effective, and user-friendly devices [1]. Such characteristics can be covered by chemical sensors and biosensors which give the opportunity to overcome drawbacks associated with traditional analytical methods, such as sample preparation, high expenses, and the requirement for qualified personnel. Amperometric biosensors are typically known for their straightforward design and rapid kinetics but also for quick response times and high efficiency [2,3]. Moreover, miniaturization for portability and mass-production make amperometric microsensors widespread in many fields of application [3,4,5,6].

Biosensors are made up of three main components: a chemical recognition system, a physicochemical transducer, and a signal processing unit. They work by converting the concentration of the analyte into a measurable signal. Biosensors, on the other hand, use a biological recognition element to detect the analyte specifically. These elements can either be receptor proteins, antibodies, polynucleotides, or enzymes. There are different types of signal transducers, such as mass-sensitive, optical, and electrochemical [7].

Electrochemistry brings numerous advantages for biosensor detection because it is not dependent on the reaction volume and can measure very small sample volumes and, most of all, very low analyte concentrations [1]. Real-time monitoring is also one of the most important features of amperometric biosensors [2,8,9,10].

In biosensor development, one of the main aims is to enhance the selectivity and specificity of devices by minimizing the signals originating from interfering molecules [11]. This turns out to be of particular importance in amperometric oxidase enzyme-based biosensors, in which the quantification of the studied analyte passes through the oxidation of Hydrogen Peroxide (HP), which takes place by applying potentials higher than 0.4 V vs. the Ag/ AgCl reference electrode [2,11,12]. Devices like these are extremely sensitive to electrochemical interferent compounds found in the matrix, with the possibility of compromising the specificity of the substrate and the selectivity of the device.

In the present paper, a glutamate biosensor design was built. This biosensor exploits the capability of glutamate oxidase (GlutOx) to recognize L-glutamate in a complex matrix specifically. As previously reported [13,14,15], the quantification of L-glutamate occurs through the oxidation of HP produced during the enzymatic reaction, as follows:

L-Glutamate + H_2_O + GluOx/FAD → α-ketoglutarate + NH_3_ + GluOx/FADH_2_

GluOx/FADH_2_ + O_2_ → GluOx/FAD + H_2_O_2_

HP, which is proportional to the glutamate concentration, can be amperometrically detected on a Pt surface when an anodic potential of +0.7 V vs. Ag/AgCl is applied [16,17] according to the following reaction

H_2_O_2_ → O_2_ + 2H^+^ + 2e^−^

The use of electrogenerated polymer films to immobilize biomolecules, both in and on, has become a popular method for producing bioanalytical devices. Moreover, electrochemical deposition enables highly reproducible and precise procedures [18].

There are two substantial issues that can impact the efficiency of a biosensor: the existence of interferents and biofouling. Actually, electroactive substances can cause interference, which is particularly troublesome during electrochemical measurements on physiological matrices.

One of the most commonly used methods to overcome these issues involves the use of thin polymer membranes. These membranes work by excluding molecules based on their size or charge. For example, membranes made from electropolymerised aminobenzenes or drop-coated Nafion allow small, uncharged molecules like hydrogen peroxide to pass through while rejecting larger interferents with higher molecular weight or charge [10].

Among different strategies, the electrodeposition of phenylenediamines is one of the most widespread methods [19,20,21]. It is a common procedure to layer polyphenylenediamines for biosensors’ design [2,11,16,22,23,24,25]. Furthermore, the ortho-phenylenediamine polymer showed the highest level of permselectivity against interferences, in particular for monitoring brain activity [2,20].

Moreover, this method simplifies or eliminates the need for sample preparation, making it easier for the biosensor to interact directly with the unprocessed matrix. Thus, this strategy can improve efficiency and accuracy.

It is now recognized that monomers such as phenylenediamines are toxic, so it is essential to focus attention on non-hazardous compounds in order to make the whole process of building biosensors sustainable.

In this study, naturally occurring compounds were utilized, as they have lower toxicity compared to OPD, and to promote the use of sustainable processes in biosensor construction. The goal was to use natural or natural-like monomers to achieve this. Although the performance of the polymers is not yet at the level of the gold standard, PPD, this effort is a significant step towards achieving green chemistry and sustainability in processes.

It has been demonstrated that also many natural phenolic compounds, with antioxidants propriety, are particularly interesting for their electrochemical activity and capacity for electropolymerization [2,11,26,27,28,29].

In the present work, two natural phenols Zingerone (ZING) (4-(4-hydroxy-3-methoxyphenyl)-butan-2-one), extracts from the rhizome of *Zingiber officinale*, and Zingerone dimer (ZING DIM) (4,4′-(6,6-dihydroxy-5,5′-dimethoxy-[1,1′-biphenyl]-3,3′-diyl)-bis-(butan-2-one)) have been used in order to evaluate the suitability for the construction of an amperometric glutamate oxidase-based biosensor.

In this work, ZING and ZING DIM polymers permeability towards both HP and Ascorbic Acid (AA), used as the archetype of the interfering species, as previously reported, was carried out [30]. Since their structures are not known, a first characterization was supposed.

## 2. Results and Discussion

### 2.1. Electrochemical Behaviour of ZING and ZING DIM by Means of Cyclic Voltammetry

Cyclic voltammetries were preliminarily carried out in order to evaluate what was the electrochemical behavior of ZING and ZING DIM and what could be the impact, if any, of the different O_2_ concentrations on the monomer solution and the resulting polymer, because it has been previously demonstrated to enhance or block the polymerization process [31].

**Figure 1:**
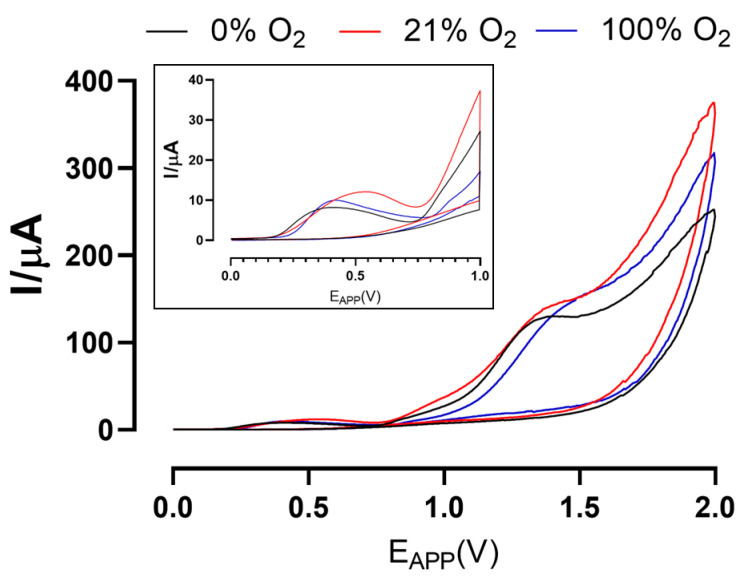
Cyclic voltammograms of ZING on Pt/Ir carried out in NaOH 0.1 M in the 0–2.0 V range of potentials, and with a scan rate of 100 mV s^−1^ in a solution saturated with nitrogen (black plot), air (red plot), and oxygen (blue plot). In the plot, the first cycle is reported. +400 mV vs. Ag/AgCl was chosen for polymerization.

In Figure 1, the first cycles of CVs of ZING are shown. From the plots, it is possible to see that the first oxidation peak occurred at +400 mV and that different percentages of oxygen in the solution did not substantially affect this value, which was used for the electrodeposition of the polymer in CPA.

**Figure 2:**
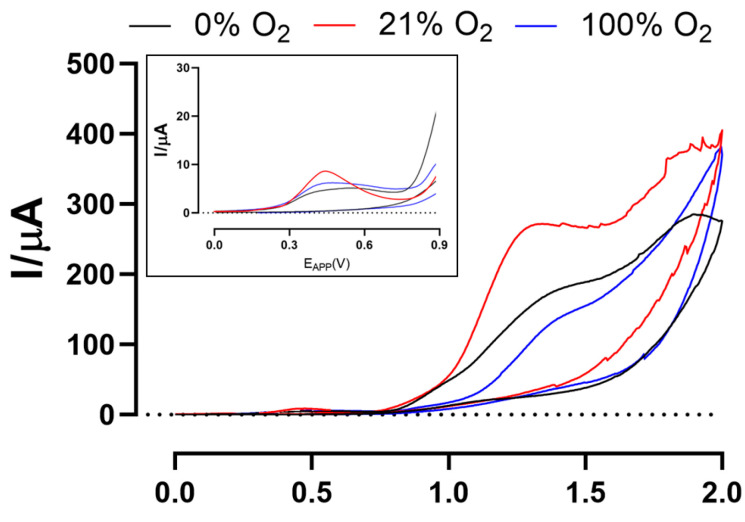
Cyclic voltammograms of ZING DIM on Pt/Ir carried out in NaOH 0.1 M in the 0–2.0 V range of potentials, and with a scan rate of 100 mV s^−1^ in a solution saturated with nitrogen (black plot), air (red plot), and oxygen (blue plot). In the plot, the first cycle is reported. +500 mV vs. Ag/AgCl was chosen for polymerization.

In Figure 2, the first cycles of CVs of ZING DIM are reported. For this compound, a first peak at +500 mV occurred, and even in this case, the different percentages of oxygen did not determine any variations. The observed potential value was set to perform polymerization by means of CPA.

As previously demonstrated for polyphenolic compounds [11], from cyclic voltammograms of ZING and ZING DIM, it is possible to evince that redox processes occurred at the Pt/Ir surface. Moreover, for both compounds (Figures S1 and S2), there was highlighted a progressive decrease of the current, visible from the second scan, which can probably be attributed to the electrode passivation due to the formation of non-conductive polymers on the electrode surface, as previously highlighted [2,26]. More, these phenomena were observed for all experimental paradigms.

On the basis of results reported by Calia et al. in 2015, in which paper it was reported that the application of low polymerization potentials resulted in an improvement in the performance of polymers derived from polyphenols, it was thus decided to proceed with the polymerization of ZING and ZING DIM by means of CPA, applying the potential of the first peak detected in the CVs.

### 2.2. Electropolymerization of ZING and ZING DIMER

The electro-polymerization of phenols is usually successfully accomplished by the use of metals such as Ti, Au, and Pt or carbon-based electrodes such as glassy carbon and graphite [26].

The polymerization process is usually executed under basic conditions that facilitate the detachment of electrons from existing phenoxide anion. The phenoxide radical could be then oxidized to quinone or react with another radical to form linear or branched oligomers and, subsequently, polymers [32].

Several factors govern the kinetic of the oxidation and the nature of polymerization, among which are the concentration of the monomeric phenolic species, the constructive characteristics of the electrode, the experimental methods of the electrolysis process, the phenols antioxidant capacity, and, finally, the pH of the reaction medium [33].

In our previous work [34], we demonstrated that natural phenol such as ZING and its symmetric dimer dehydrodizingerone (ZING DIMER) show a higher antioxidant activity than their unsaturated α-β derivatives dehydrozingerone monomer (DHZ) and dimer (DHZ DIMER) and corresponding O-methyl ZING derivatives. On this basis, and continuing our studies on the electropolymerization of natural phenols [2,11,35] such as eugenol and magnolol to obtain permselective films alternative to polyphenylenediamine, in the present work, we have focused our attention on the formation of permselective polymers starting from the monomeric units ZING and ZING DIM.

**Figure 3:**
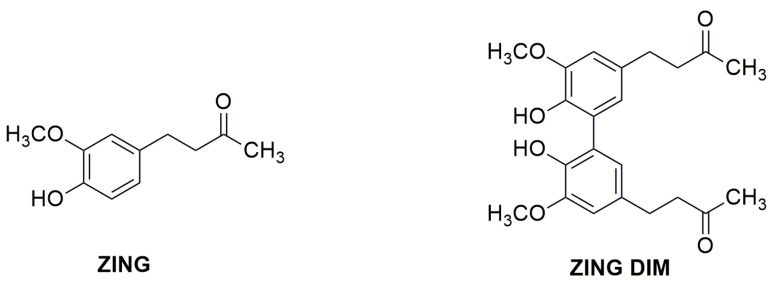
Chemical structures of Zingerone (ZING) and Zingerone dimer (ZING DIM).

C_2_ symmetric ZING DIM (Figure 3) has been reported to have generally a slightly higher antioxidant capacity than its monomer ZING by DPPH, TEAC [34], ORAC assays [36], by computational density functional theory (DFT), and by experimental lipid chain-breaking activity studies [37].

**Scheme 1:**
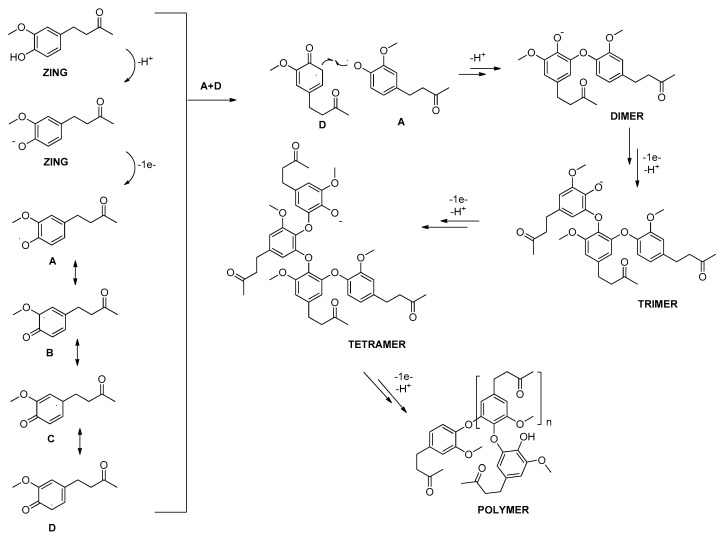
Electro-oxidation of **ZING** in basic medium and hypothetical polymerization via hetero coupling (A + D).

The antioxidant potential of ZING and ZING DIM can be attributed to the initial phenoxy radical formation and subsequent radical delocalisation on the aromatic ring to form several resonance structures [36]. In alkaline solution (pH > 9.8), ZING and ZING DIM are present as phenoxide anions. These species undergo one-electron anodic oxidation leading to the formation of phenoxy radical **A**. This radical is largely delocalized in aromatic ortho and para positions, giving rise to four radical species (**A**–**D**). Due also to the mesomeric electron-donating effect of the methoxy group, these radicals can undergo coupling radical reactions leading to oligomerization and polymerization. In Scheme 1, the hypothetical four resonance structures of ZING radical **A**–**D** radicals are reported. The oxidative hetero-coupling reactions between phenoxy radical **A** and the most reactive, from the steric point of view, radical **D** generate through various intermediate steps a polymeric structure.

**Scheme 2:**
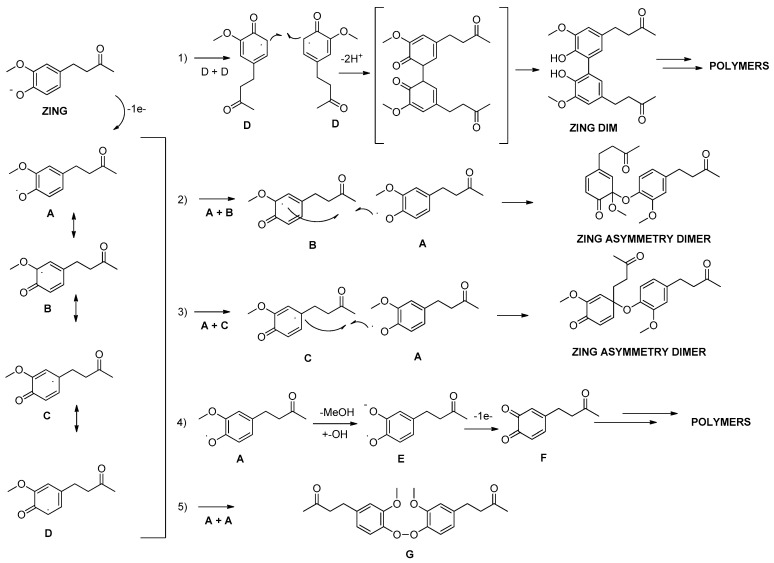
Hypothesized reactions of ZING radicals resonance forms (A–D) in alkaline medium: (1) D+D homocoupling reactions; (2) and (3) A + B and A + C heterocoupling reactions; (4) polymerization of radical **A** via loss of a MeOH molecule; (5) A + A homocoupling reaction to give peroxide **G**.

Theoretically, other coupling reactions between the several resonance forms are possible, for example, the oxidative homo-coupling reaction between two **D** radicals to form ZING DIM [34], hetero-coupling reactions between **A** and **C** or **A** and **B**, to give, as a terminal step, asymmetric dimers endowed with quinone structures and, finally, reaction between two phenoxy radicals **A,** which could lead to peroxide **G**. An additional mechanism of electro-polymerization of ZING can be supposed on the basis of previously reported eugenol electro-oxidation, the most studied natural phenol in the context of preparations of a permselective protective electrode films. The methoxy group of eugenol can induce multiple parallel reactions, such as alkaline hydrolysis, with the consequent elimination of a methanol molecule [38,39]. ZING, having in common with eugenol the guaiacyl moiety, could display similar behavior. In the oxidation of ZING, the loss of a methanol molecule from radical **A** could give rise to radical anion **E**, which in turn could be readily oxidized to quinone **F** (Scheme 2).

In the mono-electro-oxidation of ZING DIM, in alkaline medium, eight radicals can be hypothesized; however, the C_2_ symmetry axis present in the biphenyl makes the two aromatic rings indistinguishable and allows one to halve the number of hypothetical resonance forms.

**Scheme 3:**
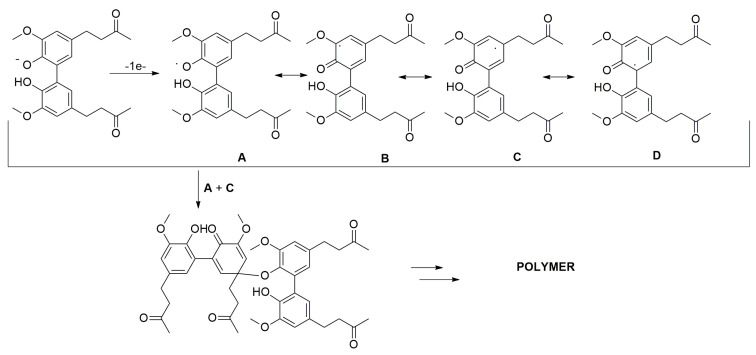
Electro-polymerization of ZING DIM under basic conditions.

The phenoxy radical, in this case, is delocalized in the sterically hindered ortho and para positions of the aromatic ring. Oxidative coupling reactions of radicals can generate different radicalic dimeric quinonic structures (e.g., **A**–**C**), which could evolve into complex polymeric structures structurally very different from those hypothesized for the ZING (Scheme 3).

In the ZING DIM, further phenoxy radical stabilization can be hypothesized due to the intramolecular interaction between the phenoxy radical itself and the hydroxyl group present in the guaiacyl structure of the second aromatic ring. This characteristic, absent in the corresponding ZING monomer and peculiar to biphenyl systems, could have a deep influence on the distribution of the various radical forms and, therefore, on the composition and geometry of the final polymer.

### 2.3. ZING and ZING DIM Polymers Responses versus HP, AA, and Ferricyanide

Since the characteristics of the polymers obtained in the present work are not present in the literature, their shielding capabilities against AA, as an archetype of interference for biosensors, and their behavior against HP, a molecule produced by the enzymatic reaction and necessary for the quantification of glutamate, were evaluated and reported as percentage variations with respect to the values obtained on the bare metal. While for AA, the variations occurring in the current monitored at a concentration of 1 mM, which is used to compare the absolute ability of polymers to block AA [30], were taken into consideration, for HP, the variations taking place in the slope values obtained from the linear regression of the calibration data were evaluated. Moreover, the influence of O_2_ % during polymerization was considered.

**Figure 4:**
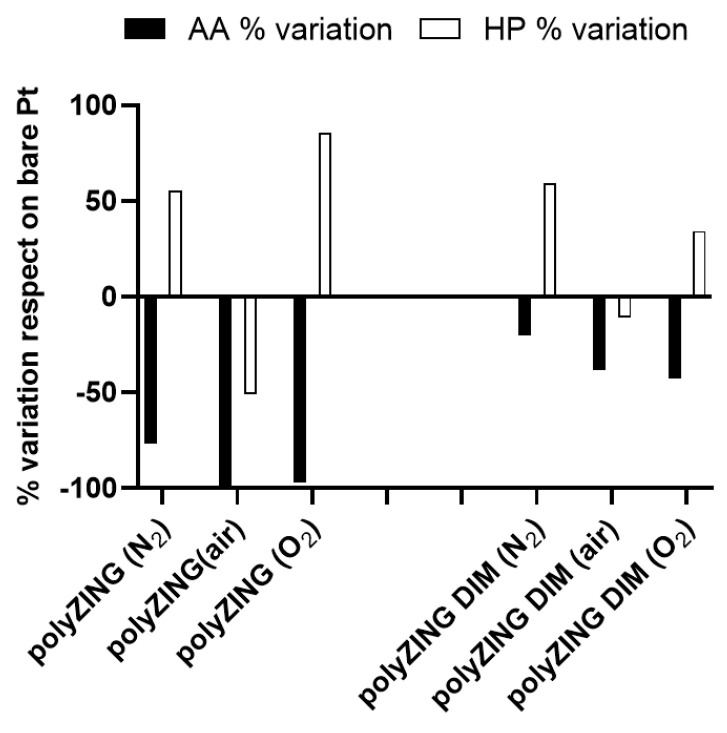
Calculated % variations responses on polyZING and polyZING DIM with respect to bare platinum. AA variations were calculated on 1 mM currents, while HP differences were determined on slopes’ value obtained by linear regression of data. % variations were calculated for polymerizations occurred in N_2_-, air-, and O_2_-saturated solutions.

As highlighted in Figure 4, both polymers showed a capability of shielding AA currents, as expected from the formation of a non-conductive polymer [2,11,30]. The polymer obtained from the ZING DIM produced a weak shielding of the interfering molecule, showing a decrease in the values of the 1 mM current, compared to those monitored on the bare platinum, ranging between 20.4% and 43%.

On the contrary, the polymer obtained from ZING demonstrated an interesting ability to block AA interference since variations ranged between 77.6 and 99.6%. The latter data, obtained when the monomer was obtained in the air-saturated solution, give results comparable with those generally obtained on the polymer obtained from OPD.

**Figure 5:**
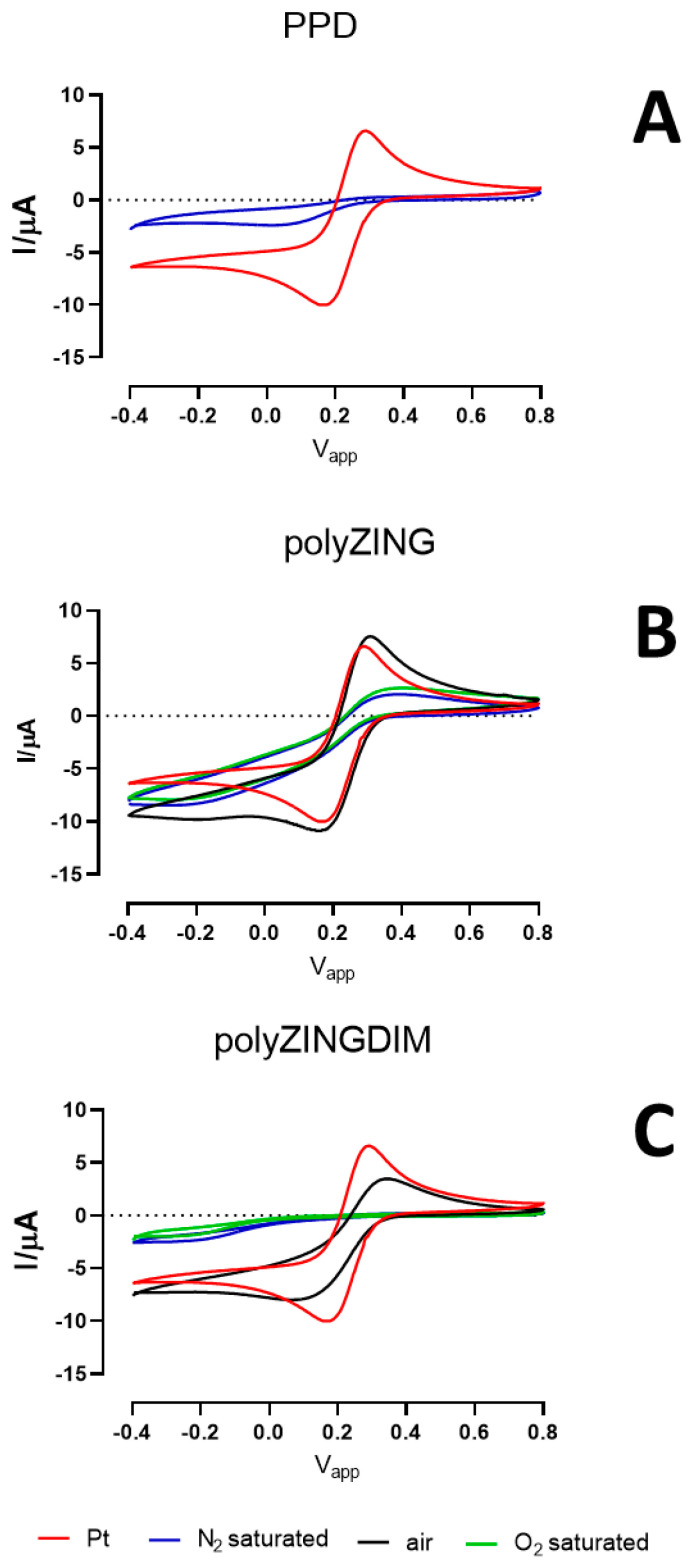
Ferro/ferricyanide couple plots obtained by cyclic voltammetry on PPD (Panel (**A**)), polyZING (Panel (**B**)), and polyZINGDIM (Panel (**C**)) are shown. Graphs are obtained on bare platinum (red line), and in N_2_- (blue plot), air- (black plot), and O_2_- (green plot) saturated conditions of polymerization. Voltammograms were obtained in 0.1 M KCl solution, in the −0.4 ÷ 0.8 V range, with a scan rate of 0.1 V/s. Currents are plotted as µA. In all experiments, the ferricyanide concentration was 0.1 M (in KCl 0.1 M).

Figure 5 displays the graphs obtained from ferricyanide (0.1 M) CVs (in 0.1 M KCl), in the presence of PPD (Panel A), polyZING (Panel B) and polyZINGDIM (Panel C) polymers. As highlighted, the reversible waves of potassium ferricyanide present on the uncoated platinum surface (red line) were entirely obscured when PPD was present but also when polyZING and polyZINGDIM were layered in N_2_- (blue line) and O_2_- (green line) saturated conditions. On the contrary, peaks from ferricyanide where conserved, with overlapping potentials and amplitude, comparable to those of the bare Pt, when ZING and ZING DIM were polymerized in air-saturated conditions.

Both polymers showed interesting behavior against HP monitoring. With the exception of the polymers obtained from the solution bubbled with air, those obtained in conditions of absence or saturation of O_2_ have shown an increased ability to monitor HP, evidenced by the percentage increase of the slope. In particular, the polymer obtained from ZING showed increases ranging from 55.6 to 85.6 when compared with bare platinum data.

The significant differences in terms of response towards H_2_O_2_ and AA shown by the polymeric films derived from **ZING** and its dimer **ZING DIM** under different degassing conditions probably depend on the geometry, thickness, and compactness of polymers and, therefore, on the chemical structure of the resulting polymers themselves. The presence of oxygen is known to inhibit radical polymerization [40] and electro-polymerization [41] by reacting with the active radicals and generating dead chain ends. In all three polymeric electro-deposition conditions—air, under pure nitrogen, and under pure oxygen—the best results, in terms of the response of the electropolymerized film, for AA and H_2_O_2,_ were obtained with ZING. It appears evident how the presence or the absence of molecular oxygen considerably influences the performances of the two polymerized compounds.

It is interesting to note that while for the electro-polymerized ZING-based sensor, the variation of shielding with respect to the standard and relative to AA is constant and close to −90%, the response relative to H_2_O_2_ is highly variable. The results obtained using ZING DIM-based polymeric films reflect the trend observed for the monomer, albeit with a generalized decrease in the permselective activity.

The AA/H_2_O_2_ selectivity of the electrode improved when the experiment was performed in O_2_ atmosphere. This can be rationalized based on the selective permeation of H_2_O_2_ over the interferences, probably due to a compound size exclusion mechanism [42].

A potentially effective approach to overcoming interference problems [43] involves the modification of the electrode surface with a self-assembled monolayer (SAM) or a polymeric film, which may be permeable to H_2_O_2_ and relatively impermeable to larger interfering species. These electrode coatings could reject interferents by unfavorable chemical, electrostatic, and/or steric interactions. However, the chemical characterization of electro-polymerized films is usually limited to infrared (IR), scanning electron microscopy (SEM), Fourier-transform infrared spectroscopy (FTIR), nuclear magnetic resonance (NMR), cyclic voltammetry, and electrochemical impedance spectroscopy (EIS) [44]. Very few are the investigation on structure determination of three-dimensional porous chemical structure of the polymers [45,46,47,48], so molecular modelling methods could certainly be useful for this purpose.

As shown in Panel A of Figure 5, the formation of the PPD polymer gave the formation of a non-conductive polymer, as widely reported in the literature [49,50,51]. As previously reported [52], this phenomenon was further highlighted by the disappearance of the oxidation and reduction peaks of the ferricyanide, which instead are clearly visible from the voltammograms obtained on bare platinum. The same behavior was displayed by polyZING (Panel B) and polyZINGDIM (Panel C) when monomers were polymerized in N_2_- (blue plot) and O_2_- (green plot) saturated conditions. Surprisingly, when ZING and ZING DIM polymerization were carried out in air-saturated conditions, the reversible peaks of potassium ferricyanide were conserved with the same potentials and amplitudes monitored on bare platinum. In the literature, the ferricyanide is used to study the electrochemical characteristics of non-conductive polymers [52]. In fact, the polymeric films presented in this work, in addition to interfering with the diffusion of ferricyanide, could also interact with it. In particular, while the data of the polymerizations of ZING and ZINGDIM carried out in N_2_- and O_2_-saturated conditions are in line with those obtained with PPD, the data obtained with the polymerization in air-saturated environment were peculiar. However, the complexity of the polymeric films, the interactions with AA, and the increased sensitivity versus HP, as described above, but also the morphologies, do not allow us to provide an unequivocal explanation. This singular behavior monitored on polymers obtained in air-saturated conditions requires further investigations necessary to clarify the nature of the polymer obtained and its electrochemical characteristics, but above all, is necessary in order to fully understand their responses towards HP.

### 2.4. Molecular Modeling Study

While in the electropolymerization of ZING DIM, three different routes for the coupling of phenoxy radical on similar sterically hindered ortho and para aromatic positions can be supposed (i.e., **A** + **C** in Scheme 3), in the case of the polymerization of the ZING monomer, a main attack of the phenoxy radical on the unsubstituted ortho carbon with consequent formation of a regular and linear polymer can be expected as described in Scheme 1 (**A** + **D**). These observations are also supported by literature data describing the electropolymerization process of similar 4-substitued natural phenols [26].

These considerations, and the very interesting characteristics regarding HP permeability data shown by the ZING polymeric film compared to that obtained by dimer polymerization, led us to concentrate our theoretical studies on this polymer.

**Figure 6:**
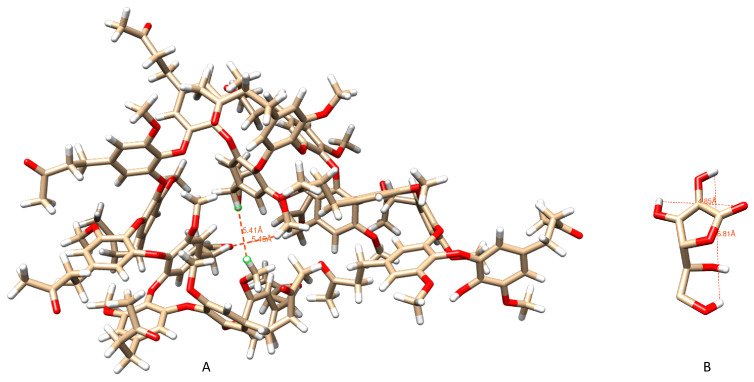
(**A**) Representation of one of the possible conformations of sixteen moieties oligopolymer structure as determined by 100 ns dynamic molecular and minimum hole diameters, and (**B**) tridimensional dimensions of the best pose of AA by docking experiment.

To elucidate the three-dimensional structure of the probable oligomer formed during the electropolymerization of the ZING, as well as its interactions with AA, a polymer consisting of 16 units was constructed. Using molecular dynamics, a 100-nanosecond simulation was conducted in the presence of an explicit aqueous solvent, resulting in 5000 frames. From the obtained structure, a representative frame was chosen to depict the average structure. The results are shown in Figure 6.

**Figure 7:**
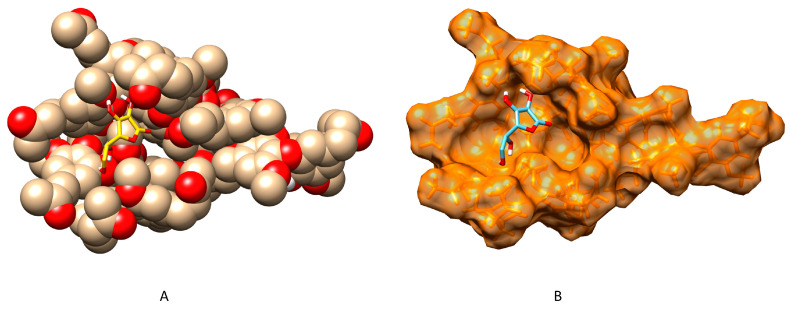
Two different representations of best pose of docking between AA and sixteen ZING moieties oligomer. (**A**) Sphere model and (**B**) surface model.

In this model, a tunnel in the central part of the structure can be observed. The dimensions of the interference compound AA, obtained from the best pose, are approximately 6.81, 4.85 Angstroms (height, width) (Figure 6B). It is noteworthy that the maximum hole diameter (5.46 × 5.41 Angstroms) (Figure 6A) of the polymer is large enough to allow permeability to HP but could pose a significant steric barrier to a relatively larger interferent like ascorbic acid. Already from the early stages of the dynamics, it is observed that the long polymer chain, initially linear, tends to fold upon itself, finding intramolecular interactions that partially induce stability. The obtained structure from the dynamics simulation was also used as a starting point to perform docking calculations necessary to observe all possible interactions with AA. All docking tests were performed considering a 40 × 40 × 40 grid centered on the oligomer, with the center located close to the tunnel. As a result, a large number of docking sites were evaluated, treating the docking active site as a rigid system and the AA as flexible. It can be observed that ascorbic acid interacts with the site in proximity to the tunnel, with a docking score estimating free energy of binding of −4.41 Kcal/mol and an inhibition constant of 585.44 µM. This interaction was observed in 54% of the docking runs near the tunnel site (Figure 7).

**Figure 8:**
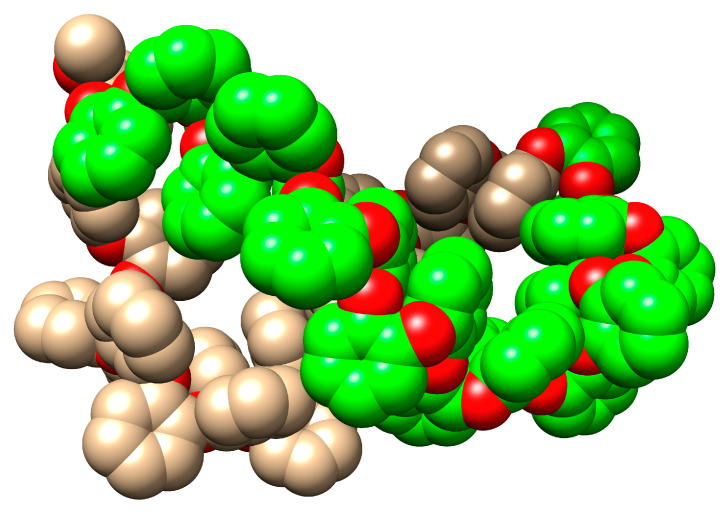
Representation of one of the possible minimized conformations of forty moieties oligopolymer structure as determined by 100 ns dynamic molecular. In the figure, only the aromatic portion of the oligomer is mainly represented, with the carbon atoms of 20 out of the 40 oligomeric units of the strand shown in green, in order to better visualize the structural conformation of the backbone.

Preliminary results from molecular dynamics simulations on a 40-unit ZING template model in explicit solvent indicate that the growth of the polymer is likely to proceed three-dimensionally in a helix-sense-selective fashion. (Figure 8) [48].

It is important to note that the modelling approach provides only an estimation of interactions between the polymer and interfering species, as it does not consider a wide range of interfering parameters such as concentration, interactions with other species, interactions with water molecules, delivery system, membrane permeability, biocompatibility, and bioavailability. Nevertheless, the interactions highlighted in this study can serve as an additional tool to discover new polymers based on natural phenols with high permselective properties, reducing research time and costs. We expect to further develop this aspect in the future by applying computational techniques to obtain structural information on the electropolymerization of various natural monomeric phenols.

### 2.5. Scanning Electron Microscopy Study of Polymers

**Figure 9:**
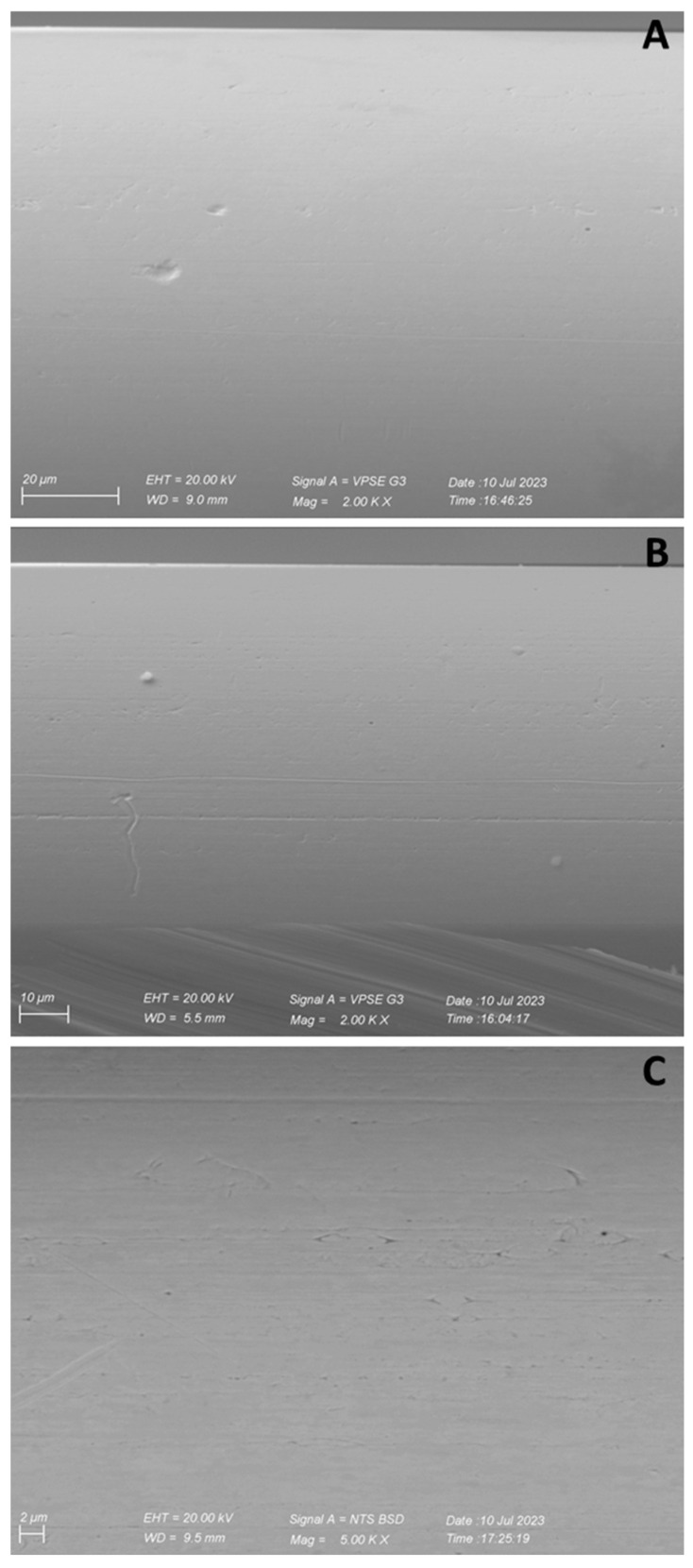
Scanning electron micrographs of polyZING obtained in N_2_- (Panel (**A**)), air- (Panel (**B**)), and O_2_-saturated (Panel (**C**)) solution, 2000× of magnification.

**Figure 10:**
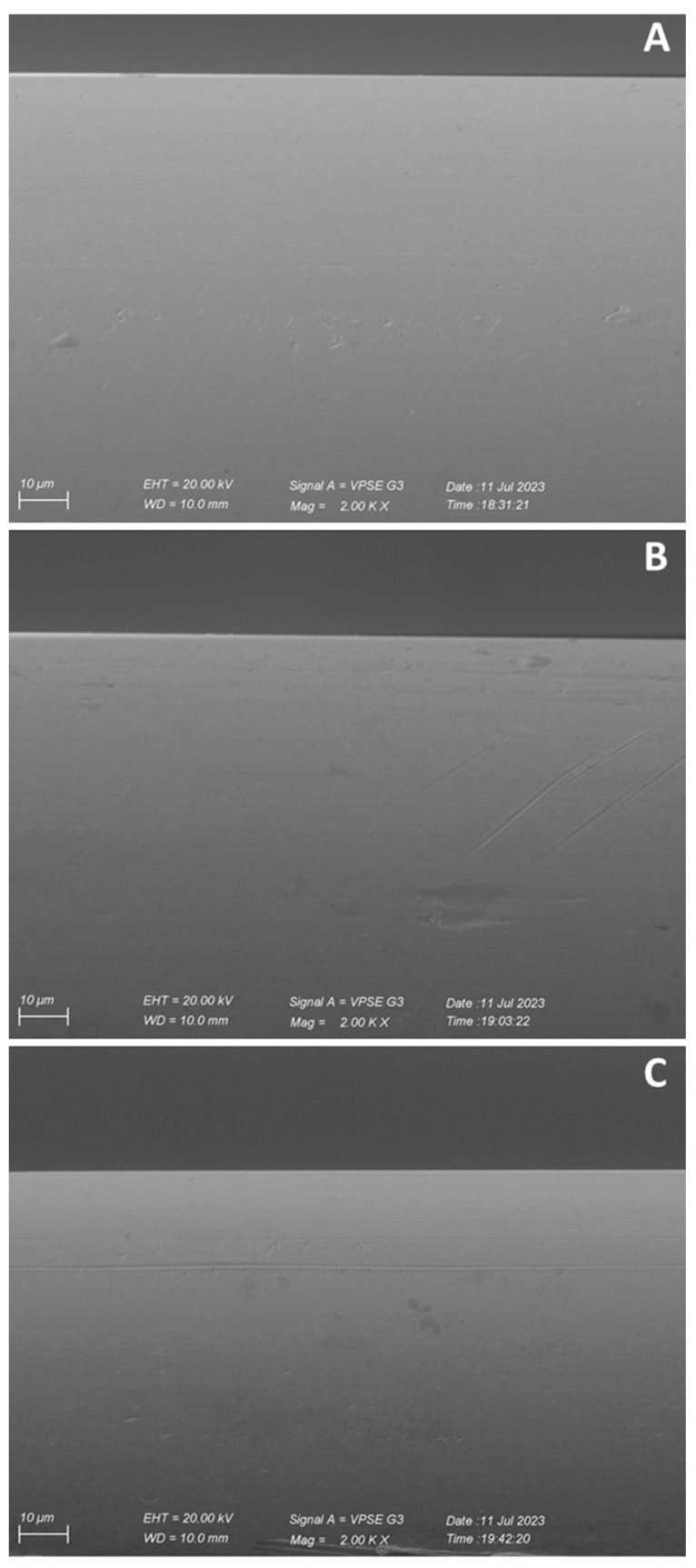
Scanning electron micrographs of polyZING DIM obtained in N_2_- (Panel (**A**)), air- (Panel (**B**)), and O_2_-saturated (Panel (**C**)) solution, 2000× of magnification.

As shown in Figure 9 and Figure 10, the process of electropolymerization resulted in a regular, ordinate, and compact surface, for both polyZING and polyZING DIM. Moreover, as highlighted in Figure 9 Panel B and in Figure 10 Panel B, despite the deposition of the polymer, it is still possible to see the imperfections of the bare platinum generated during the construction of the sensor, highlighting the probable formation of a polymer of relative thickness.

From microphotographs, it is not possible to extrapolate any further information about the morphology of the deposited polymer.

Moreover, results obtained with ferricyanide, AA, and HP suggest that behind the apparent morphological similarity, there are profound functional differences; therefore, further studies are in progress in order to better evaluate the structure of the obtained polymer, even to better elucidate the interesting features regarding the increased monitoring of HP.

### 2.6. In Vitro Characterization of Glutamate Biosensors

Based on the particular characteristics highlighted on both polyZING and polyZING DIM (Figure 10), related to good AA shielding and increased HP monitoring, glutamate biosensors were built using the polymers mentioned above and compared with the previously described PPD-based glutamate biosensors [13,14,53]: actually, PPD is the most commonly used polymer for amperometric biosensors because of its peculiarities [2,20].

As exposed in Figure 10, kinetic parameters, such as V_MAX_ and K_M_ (Panel A and B), were calculated by means of a nonlinear fitting of data derived from calibrations ranging from 0 to 50 mM of glutamate. Meanwhile, LRS, the analytical parameter, was obtained by means of a linear regression of data in a range between 0 and 400 µM of glutamate (Panel C). The plots show results from different glutamate biosensor designs. In particular, purple bars refer to PPD-based biosensors, while blue and green bars indicate data from polyZING- and polyZING DIM-based biosensors, respectively.

**Figure 11:**
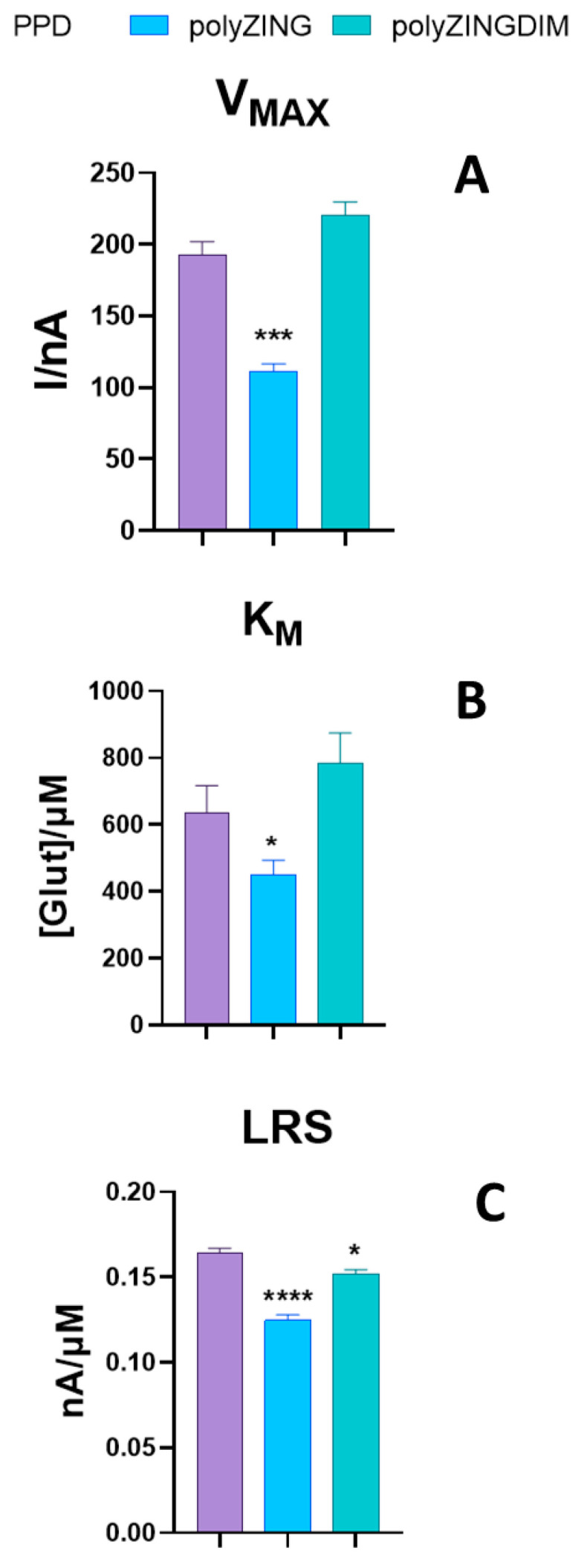
Bar plot of the variation of kinetic parameters as V_MAX_ (Panel (**A**)) and K_M_ (Panel (**B**)) and for the Linear Region Slope (LRS) (Panel (**C**)) for different biosensor designs (n = 4) based on different polymers: Pt_c_/PPD/PEI(1%)_2_/GlutOx_5_/TEG(0.1%) (purple bars); Pt_c_/polyZING/PEI(1%)_2_/GlutOx_5_/TEG(0.1%) (blue bars); Pt_c_/polyZINGDIM/PEI(1%)_2_/GlutOx_5_/TEG(0.1%) (green bars); Pt_c_: 1 mm Pt cylinder; PPD: ortho-phenylenediamine polymer; polyZING: ZING-based polymer; polyZING DIM: ZING DIM-based polymer; GlutOx: L-glutamate oxidase; PEI: polyethyleneimine, TEG: triethylene glycol. Values are given as mean ± SEM.* *p* < 0.05 vs. PPD; *** *p* < 0.01 vs. PPD; **** *p* < 0.001 vs. PPD.

As shown in Figure 11, Panel A, PPD-based biosensors displayed a V_MAX_ equal to 192.6 ± 9.4 nA. This value results in being in line with the ones previously published. Contrary to expectations, the polyZING-based biosensor showed a lower V_MAX_ (*p* < 0.001 vs. PPD) equal to 111.4 ± 5.1 nA, while the polyZINGDIM-based biosensor exhibited a V_MAX_ value of 220.7 ± 9.0 nA, resulting as not statistically different from the PPD value. About the K_M_ results (Panel B), only polyZING-based biosensors showed a statistical difference (*p* < 0.05) from PPD-based with a value equal to 451.5 ± 41.2 µM. As for LRS (Panel C), both polyZING and polyZINGDIM showed values statistically different from PPD, equal to 0.125 ± 0.003 (*p* < 0.001 vs. PPD) and 0.152 ± 0.003 (*p* < 0.05 vs. PPD) nA/µM, respectively.

In Table S1, LOD and LOQ values for all the designs are reported. It is possible to highlight that PPD-based biosensors showed the best LOD and LOQ values, 0.237 ± 0.001 and 0.791 ± 0.002 µM, respectively. polyZING- and polyZINGDIM-based biosensors displayed higher values (*p* < 0.0001) when compared with PPD-based biosensors (data are reported in Table S1).

In Table S1, values of 1 mM of AA are also reported, which can be used to compare the absolute ability of polymers to block AA [30]. As shown in the table, the PPD-based design turned out to be the best in terms of AA shielding showing a 1 mM AA value equal to 0.398 ± 0.163 nA. On the contrary, other designs resulted in being less efficient (*p* < 0.0001 vs. PPD) in shielding AA, as they showed values equal to 5.307 ± 0.054 and 6.750 ± 0.687 nA, respectively, for polyZING based- and polyZINGDIM based-design.

The promising results obtained by the AA shielding and HP-increased monitoring on polyZING and polyZINGDIM led us to think that these polymers could improve the performances of a biosensor for glutamate detection.

In a preliminary series of experiments, given the peculiar characteristics of the polymers against HP, the kinetic performances of the biosensors in the absence of the enzymatic booster PEI were investigated. V_MAX_ and K_M_ values for polyZING- based biosensors were 36.6 ± 2.3 nA and 326.8 ± 73.2 µM, while for polyZINGDIM-based biosensors, they were 45.7 ± 2.8 nA and 368.8 ± 76.8 µM. Linear region slopes were 0.068 ± 0.002nA/µM and 0.076 ± 0.002 nA/µM, respectively. Data showed that the peculiar increase in the hydrogen peroxide reading was not maintained for both polymers.

From biosensor data characterization, it was evident that both the features inherent in AA and HP were not maintained in the presence of all biosensor components, especially when data were compared with the biosensor were based on PPD polymer, which is one of the most commonly used in amperometric biosensors because it is able to block interferences from several compounds, such as ascorbic acid, but at the same time, is able to save the activity of the enzyme, improving its stability and sensitivity. The observation of the enzymatic kinetic data showed that polyZINGDIM is similar to the PPD-based design, while polyZING was clearly showed not to facilitate the enzymatic activity, despite revealing a better enzyme–substrate affinity.

The analytical parameters of the biosensors related to LRS are superimposable to those observed for the kinetic parameters, while LOD and LOQ revealed that the PPD-based biosensor is more sensitive in the detection and quantification of glutamate.

However, although the data obtained from glutamate biosensors fell short of initial expectations, an interesting increase in the HP reading was observed for the first time on polymers obtained from ZING and ZING DIM. This observation makes these polymers excellent candidates for future studies aimed at the development of electrochemical devices for the detection of HP (also based on other types of transducers).

However, further studies are currently underway to try to understand which molecular mechanisms underlie the increased HP monitoring.

Moreover, additional experiments are planned to study in more depth whether the characteristics observed on the above-mentioned polymers can be maintained in the presence of other enzymes or with the deposition of other components necessary for the construction of biosensors. Furthermore, studies on the conservation of these biosensors at very low temperatures are planned [14] to verify the consistency, and, possibly, the improvements in the features of the biosensor designs presented in the present work.

## 3. Experimental

### 3.1. General Procedures and Reagents for Compounds’ Synthesis

Unless otherwise specified, starting materials and reagents were obtained from Sigma Aldrich (Munich, Germany) and used without further purification. Melting points were determined on a Büchi 530 instrument (Flawil, Switzerland) and are uncorrected. All ^1^H NMR and ^13^C NMR spectra were recorded in CDCl_3_ solution at 600 MHz and 150 MHz, respectively, using a 600 MHz NMR spectrometer Bruker Avance III HD (Palo Alto, CA, USA). Chemical shifts are in ppm (δ); multiplicities are indicated by s (single), d (duo), t (triple), q (quad), m (multiple), or dd (double of double). Elemental analysis for C, H, and O were performed on a PerkinElmer 240 C elemental analyzer. Flash chromatography was carried out with silica gel 60 (230–400 mesh) (VWR, Radnor, AF, USA) eluting with an appropriate solution in the stated v:v proportions. All reactions were monitored by analytical thin-layer chromatography (TLC) with 0.25 mm thick silica gel plates (60 F 254) (Sigma Aldrich, Munich, Germany). The purity of all new compounds was judged to be >98% by 1H-NMR spectral determination. ZING was purchased from E. Merk (Darmstadt, Germany) and was used without further purification. ZING DIM was prepared according to [34] with slight modifications. In-silico analysis: model compounds ascorbic acid and monomer zingerone acid were constructed with standard bond lengths and angles from the fragment database with Gaussian 09 [54], and representative minimum energy conformations of the compounds were optimized using the ab initio quantum chemistry program with method B3LYP/6-311G basis set. Visual analysis was performed with Gaussianview version 5 [55]. Molecular Dynamics: the starting oligopolymers (sixteen and forty units, were prepared with Leap e antechamber program of AMBER18 [56]. Molecular Dynamics production simulations were carried out using the PMEMD GPU version included in the AMBER18 suite of programs, with the GAFF force field, after careful relaxation of the system using 13 phases of minimization and equilibration protocols. The oligomers as well as all the water molecules of the structure, were surrounded by a periodic box of TIP3P32 water molecules that extended 10 Å from the polymer. Langevin dynamics was used to control the temperature (300 K). Periodic boundary conditions were applied to simulate a continuous system. RESP charges were derived by using the Gaussian 09 and antechamber program. In the MD simulation protocol, the time step was chosen to be 2 fs, and the SHAKE algorithm was used to constrain all bonds involving hydrogen atoms. A nonbonded cutoff of 8.0 Å was used for the production dynamics. UCSF—Chimera was used as a visualization program for visualizing, animating and analyzing large biomolecular systems using 3D graphics and integrated scripting [57]. Finally, the production step was carried out under the equilibrium conditions, and the system was subjected to 100 ns MD simulation for the polymer. One hundred ns of the trajectories from each case were considered for statistical analysis. The trajectories were analyzed using the PTRAJ module of AMBER. Computational modelling experiments were carried out on a HP8100 PC and an EXXACT Tensor Workstation TWS-1686525-AMB with GPU and parallel capability. Docking studies: AutoDockTools 1.5.4 [58] and AutoDock-GPU ver.1.5 docking programs [59]. Hydrogen atoms were added using the ADT module. We used the Gasteiger charges of Autodock for the ligands and oligomers. The structures were docked using the Lamarckian genetic algorithm (LGA) defined through a grid centered 30.382 27.284 28.239, with 40, 40, 40 grid point in X, Y, Z dimensions, respectively. We used the default grid spacing (0.375 Å) and performed 100 docking runs, treating the docking active site as a rigid molecule and the ligands as flexible, i.e., all non-ring torsions were considered active.

### 3.2. Synthesis of ZING DIM

[4,4′-(6,6′-dihydroxy-5,5′-dimethoxy-[1,1′-biphenyl]-3,3′-diyl)bis(butan-2-one)] (ZING DIM).

A solution of methyltributylammonium permanganate (MTBAP) (0.65 g, 2.00 mmol) [34] in dry dichloromethane (10 mL) was added dropwise to a solution of ZING (0.77 g, 4 mmol) in dry dichloromethane (15 mL) at room temperature under N_2_. The reaction mixture was stirred for 1 h at room temperature and then, a Na_2_S_2_O_5_ aqueous solution (30 mL) was added. The organic layer was then separated, washed with water, and dried (Na_2_SO_4_). The solvent was evaporated under reduced pressure, and the solid was purified by flash chromatography using a 1:2 mixture of ethyl acetate:petroleum ether as eluent (0.48 g, 63%); mp 85–86 °C; ^1^H NMR δ ppm 2.17 (s, 6H), 2.78–2.90 (series of m, 8H), 3.93 (s, 6H), 6.00 (bs, 2H), 6.74 (d, J = 2.0 Hz, 2H), 6.75 (d, J = 2.0 Hz, Ar, 2H); ^13^C NMR δ ppm 29.53, 30.14, 45.48, 56.14, 110.68, 122.72, 124.40, 132.91, 140.95, 147.21, 208.12; Anal. Calcd for C_22_H_26_O_6_: C, 68.38; H, 6.78; Found: C, 68.48; H, 6.73.

### 3.3. Chemicals and Solutions

All chemicals were of analytical grade or higher purity and were from Merk Life Science S.r.l. (Italy). Solutions were obtained in bidistilled deionized water. Ascorbic acid (AA), hydrogen peroxide (H_2_O_2_), o-phenylenediamine (oPD), zingerone (ZING), sodium hydroxide (NaOH), polyethileneimine (PEI), triethylene glycol (TEG), potassium chloride (KCl) and ferrycianide [K _3_Fe(CN)_6_] were from Merk Life Science S.r.l. (Italy) while dimer of ZING (ZING DIM) was synthesized as described in paragraph 3.2. The phosphate-buffered saline solution (PBS, 0.05 M) was prepared with the following composition (expressed in M concentrations): 0.15 NaCl, 0.05 M NaH_2_PO_4,_ and 0.04 M NaOH (pH 7.4). ZING and ZING DIM polymerizations were performed in NaOH (100 mM). The OPD monomer (300 mM) was prepared in PBS, whereas ZING and ZING DIM were obtained in NaOH (100 mM) and prepared immediately before use. Stock solutions of H_2_O_2_ (100 mM), AA (100 mM), and Glutamate (1 M and 10 mM) were prepared in water immediately before use. While AA solution was kept at −20 °C when not used, other solutions were stored at 4 °C. Teflon^®^-insulated platinum (90% Pt, 10% Ir; Ø = 125 μm) was from Advent Research Materials (Eynsham, England). Glutamate oxidase (GlutOx, 400 U/mL) [60] was kindly donated by Enzyme-Sensor Co., Ltd (Tsukuba, Japan). 

### 3.4. Platinum Microsensors and Glutamate Biosensor Fabrication and Characterization

**Figure 12:**
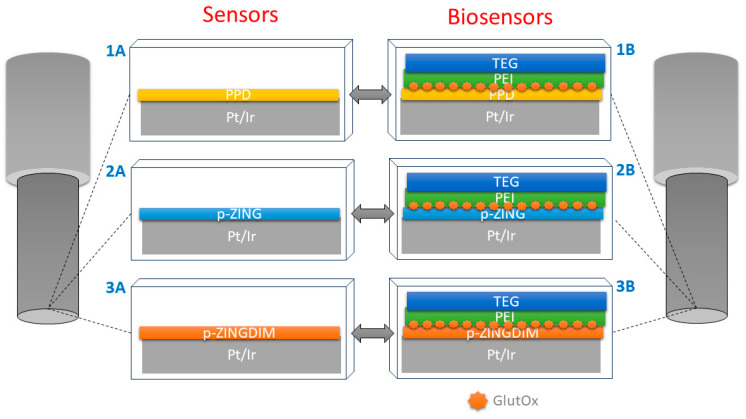
Schematic representation of polymerized Pt-based microsensors (left image) on which polymers polyZING (2A) and polyZINGDIM (2B) were layered, and biosensors were built. Pt/Ir: platinum/iridium cylinder (1 mm length, 125 µm Ø); p-ZING: polymer derived from zingerone; p-ZINGDIM: polymer derived from zingerone dimer; p-OPD: poly-o-phenylenediamine; PEI: polyethyleneimine; GlutOx: glutamate oxidase; TEG: triethylene glycol.

As highlighted in Figure 12, all electrodes had the same cylindrical geometry, obtained from the exposition of 1 mm of bare metal by removing the Teflon^®^ insulation from the 125 µm Ø platinum wire. Briefly, a portion of 3 mm of wire was obtained. From one edge, 3 mm of Teflon was removed in order to allow the soldering of the wire to the connector. On the other edge, 1 mm of metal was exposed in order to obtain the active surface to modify. HP and AA calibrations were initially performed on bare platinum, in the ranges 0–100 µM and 0–1000 µM, respectively. Preliminarily, cyclic voltammetries were performed on ZING and ZING DIM, in order to evaluate what was the working potential to be used for the electropolymerisation. As previously published [2,11], CVs were performed for 10 cycles with the monomers dissolved in NaOH (0.1 M, pH = 12.9) in a voltage range of 0 and 2.0 V and with a scan rate of 100 mVs^−1^ (Figures S1 and S2).

As shown in the insets, the oxidation peak for ZING was set at +400 mV, while for ZING DIM, it was +500 mV, both vs. the Ag/AgCl reference electrode.

For ZING and ZING DIM polymerizations, at selected oxidation peaks, oxygen influence was tested. Actually, monomers’ solutions were prepared powders in NaOH (0.1 M) bubbled with ultrapure N_2_ or O_2_, or air. Solutions were then shaken for 5 min into a closed tube, and then poured into a beaker where bare metal electrodes were immersed.

Polymerizations of ZING and ZING DIM were carried out by means of constant potential amperometry (CPA) in a solution of 0.1 M NaOH (pH = 12.9) for 10 min, as previously reported [11].

After each polymerization of ZING and ZING DIM, HP and AA calibrations, with the same scheme as on bare metal, were performed in fresh PBS in order to evaluate polymerized sensors’ behavior towards AA and HP. The percentage variations with respect to the bare metal monitoring were evaluated (Figure 4). As regards HP, the variations of the linear regression data slopes were evaluated, while for AA, the variations of the current monitored at the concentration of 1 mM were considered.

The electrochemical behavior of all polymers was studied by means of ferricyanide (0.1 M) cyclic voltammetries, that were performed in 0.1 M KCl in the potential range from −0.4 to 0.8 V (vs. Ag/AgCl) with a scan rate of 0.1 V/s.

Given the interesting results obtained (Figure 4), PPD-, polyZING-, and polyZINGDIM-based glutamate biosensors were constructed as follows.

As previously reported [13,14,53], PPD-based biosensors (n = 4) were constructed. In brief, at day 0, a PPD polymer was obtained on a 1 mm cylinder of Pt/Ir, from a 330 mM solution of OPD, previously degassed for 15 min with pure N_2_, and by applying +0.7 V for 30 min. Thus, two layers of PEI (1%) were deposited, and then 5 layers of GluOx were stratified, with 5 min intervals between each layer. Finally, the biosensor was rapidly immersed in a TEG solution (0.1%) and let dry for 30 min, obtaining the following design [14]:

Pt_c_/PPD/PEI (1%)_2_/GlutOx_5_/TEG (0.1%)

At the end of the procedures, biosensors were rinsed in pure water and put in fresh PBS and left overnight under the applied potential of +0.7 V vs. Ag/AgCl to allow the baseline current to stabilize.

The same protocol was used for ZING- and ZING DIM-polymer-based biosensors (n = 4 for each design). In these cases, the polymer deposition occurred as described above, obtaining the following designs:

Pt_c_/Poly-ZING/PEI (1%)_2_/GlutOx_5_/TEG (0.1%)

Pt_c_/Poly-ZING-DIM/PEI (1%)_2_/GlutOx_5_/TEG (0.1%)

After overnight-current stabilization, on Day 1, biosensors were put in 20 mL of fresh PBS and exposed to increasing concentrations of glutamate, ranging between 0 and 50 mM, obtained by injecting known volumes of Glut stock solutions (10 mM and 1 M) (Figures S3–S5). On the same biosensors, calibration with AA (100 mM) was performed, exposing them to 250, 500, and 1000 µM concentrations to evaluate the AA shielding (Table S1).

### 3.5. Instrumentation and Software

For electrochemical procedures, a conventional three-electrode cell was used, which comprised a beaker with 20 mL of fresh PBS, four working electrodes represented by glutamate biosensors, an Ag/AgCl (3M) electrode (Bioanalytical Systems, Inc., West Lafayette, IN, USA), and a large stainless-steel needle as auxiliary electrode. A four-channel potentiostat (eDAQ Quadstat, e-Corder 410, eDAQ Europe, Poland) was used for all electrochemical procedures. All potentials were applied against the aforementioned reference electrode.

### 3.6. Statistical Analysis

Biosensors’ currents were plotted against Glut concentrations. First, a nonlinear fitting with a Michaelis–Menten equation for biosensors’ data was performed on the total concentration range (0–50 mM) to extrapolate kinetics parameters, such as V_MAX_ and apparent K_M_ (respectively expressed as nA and µM), while the analytical parameter, such as Linear region slope (LRS), was evaluated at low concentrations (0–400 µM), performing a linear regression on obtained data and reported as nA/µM. Currents were expressed in nanoamperes (nA) and used as baseline-subtracted values ± standard error of the mean (SEM). The evaluation of AA shielding involved the analysis of the current recorded at 1.0 mM of AA in the electrochemical cell, as previously discussed [50].

Statistical significance (P values) between groups was assessed using ANOVA with multiple comparisons by means of GraphPad Prism 9.5.1 v software.

The limit of detection (LOD) and quantification (LOQ) were calculated from the standard deviation (σ) of the response and the LRS of the calibration curve as follows [61]

LOD = 3 σ/LRS

LOD = 10 σ/LRS

### 3.7. Scanning Electron Microscopy (SEM) Study

To conduct SEM analysis, the samples were mounted on carbon stubs and scanned without any pre-treatment with a Zeiss EVO LS10 Environmental Scanning Electron Microscope (Oberkochen, Germany), in low-vacuum mode (chamber pressure 10 Pa) coupled with a VPSE detector and a BSD detector.

## 4. Conclusions

The results obtained in the present work have demonstrated that the use of polyphenolic molecules of natural origin, such as ZING and ZING DIM, could be a more sustainable alternative for obtaining polymeric films for the construction of amperometric biosensors, in particular, for polyZING, due to its characteristic ability to shield interfering species eventually present in the matrices under study. The peculiar characteristic concerning the increase of HP monitoring is a point to be further investigated, given the importance of its production during the enzymatic reaction, with the aim of improving the analytical performances of the first generation amperometric biosensors.

## Supplementary Materials

The following supporting information can be downloaded at: https://www.mdpi.com/article/10.3390/molecules28166017/s1.
