# Supplementary material for: Sustainable Electropolymerization of Zingerone and Its C2 Symmetric Dimer for Amperometric Biosensor Films
Source: Molecules. 2023 Aug 11;28(16):6017. doi: 10.3390/molecules28166017 (PMC10459948; doi:10.3390/molecules28166017)
Supplement: Supplementary file 1 [file molecules-28-06017-s001.zip › molecules-2535108-supplementary.pdf]

Sustainable Electropolymerization of Zingerone and Its C2 Symmetric Dimer for Amperometric Biosensor Films

Myriam Caval[1], Maria Antonietta Dettori[2], Davide Fabbri[2]*, Roberto Dallocchio[2], Alessandro Dessì[2], Salvatore Marceddu[3], Pier Andrea Serra[4], Gaia Rocchitta [4]*, Paola Carta[2]

[1] Dipartimento di Scienze Biomediche, Università Degli Studi di Sassari, Sassari 07100, Italy
[2] Istituto di Chimica Biomolecolare, Consiglio Nazionale Ricerche, Sassari 07100, Italy
[3] Istituto di Istituto Scienze delle Produzioni Alimentari, Consiglio Nazionale Ricerche, Sassari 07100, Italy
[4] Dipartimento di Medicina, Chirurgia e Farmacia, Università Degli Studi di Sassari, Sassari 07100, Italy
* Correspondence: grocchitta@uniss.it (GR); Davidegaetano.fabbri@cnr.it (DF)

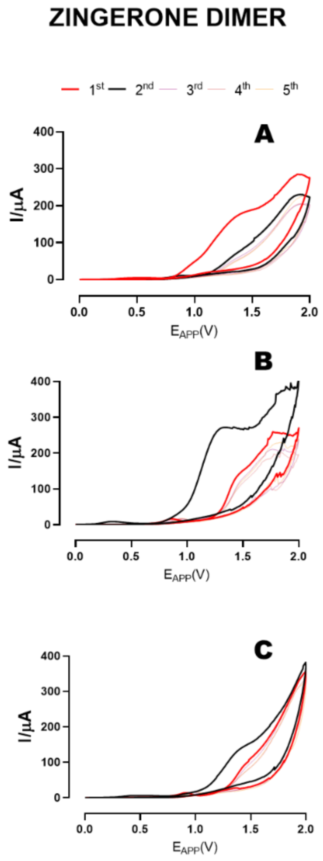

Figure S1: Cyclic voltammetries of ZING carried out in different conditions: in N_2- (Panel A), air- (Panel B) and O_2- saturated (Panel C) monomer solution. ΔE= 0÷2.0 V, scan rate: 100 mVs^{-1}. 1 to 5 cycles are reported.

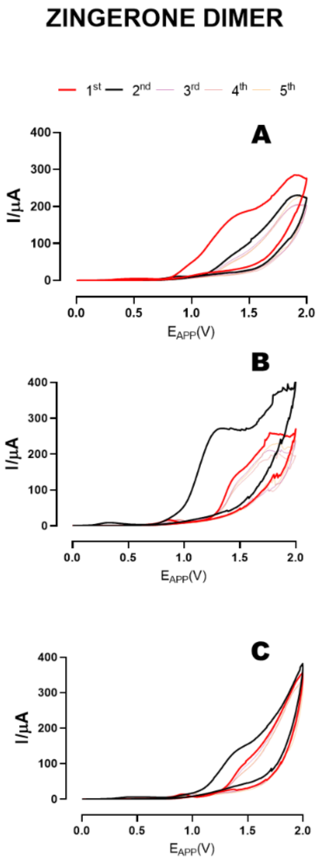

Figure S2: Cyclic voltammetries of ZING DIM carried out in different conditions: in N_2- (Panel A), air- (Panel B) and O_2- saturated (Panel C) monomer solution. $\Delta E = 0 \div 2.0$ V, scan rate: 100 mVs^{-1}. 1 to 5 cycles are reported.

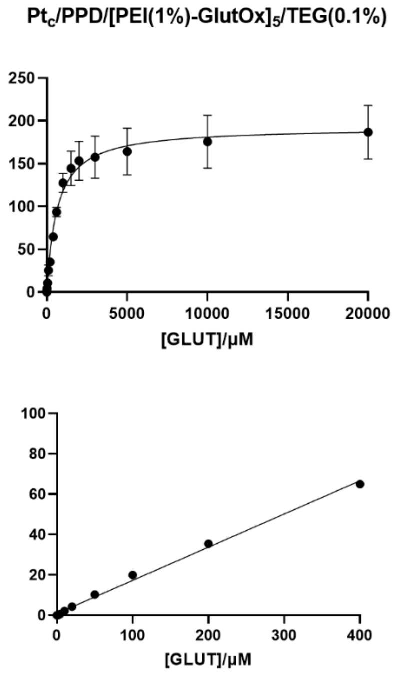

Figure S3: Michaelis-Menten kinetics plot (Panel A) ranging from 0 to 50 mM and linear regression curve (Panel B) ranging between 0-400 μM of PPD-based glutamate biosensor design at Day 1. Pt$_c$: Pt cylinder 1 mm long, 125 μm diameter; PPD: polyortho-phenylenediamine; PEI: polyethyleneimine; GlutOx: Glutamate Oxidase; TEG: triethyleneglycol. The subscript number indicates the number of dipping steps and in brackets, the concentration of the component.

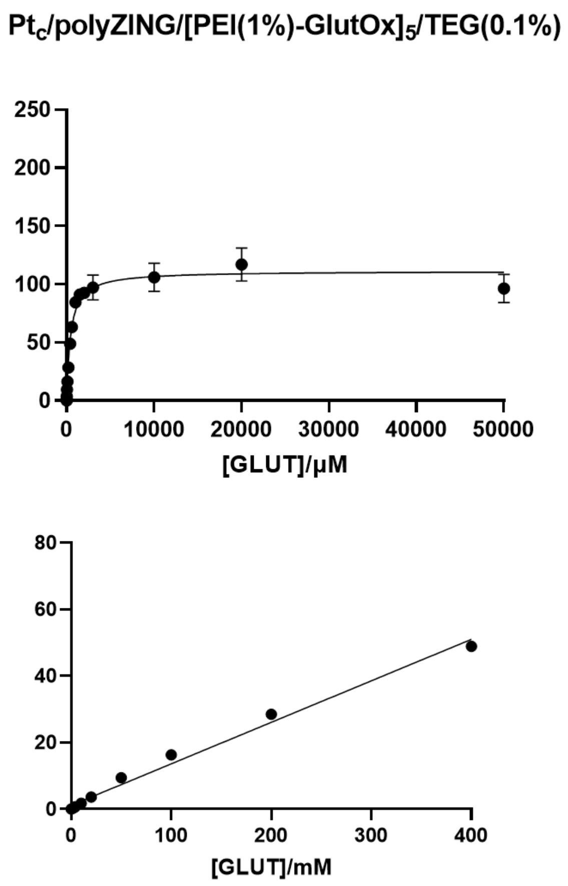

Figure S4: Michaelis-Menten kinetics plot (Panel A) ranging from 0 to 50 mM and linear regression curve (Panel B) ranging between 0-400 μM of polyZING-based glutamate biosensor design at Day 1. Pt$_c$: Pt cylinder 1 mm long, 125 μm diameter; polyZING: polymer from ZING; PEI: polyethyleneimine; GlutOx: Glutamate Oxidase; TEG: triethyleneglycol. The subscript number indicates the number of dipping steps and in brackets, the concentration of the component.

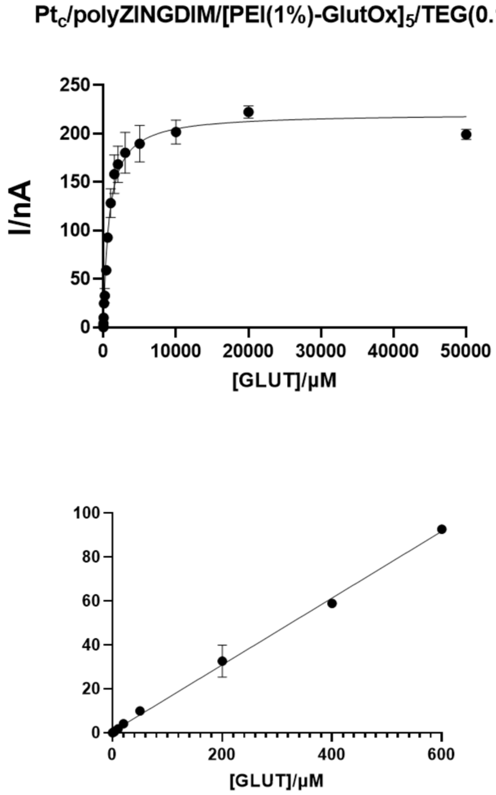

Figure S5: Michaelis-Menten kinetics plot (Panel A) ranging from 0 to 50 mM and linear regression curve (Panel B) ranging between 0-600 µM of PPD-based glutamate biosensor design at Day 1. Pt_c: Pt cylinder 1 mm long, 125 µm diameter; polyZINGDIM: polymer from ZING DIM; PEI: polyethyleneimine; GlutOx: Glutamate Oxidase; TEG: triethyleneglycol. The subscript number indicates the number of dipping steps and, in brackets, the concentration of the component.

Table S1: In vitro parameters of three different glutamate biosensor designs (n=4) at Day 1 in terms of Michaelis–Menten kinetic parameters (V_{MAX} and K_M) and analytical parameters (Linear Region Slope –LRS, LOD and LOQ) for each design. PPD-based: Pt_c/PPD/PEI(1%)$_2$/GluOx$_5$/TEG(0.1%); polyZING-based: Pt_c/polyZING/PEI(1%)$_2$/GluOx$_5$/TEG(0.1%); polyZINGDIM-based: Pt_c/polyZINGDIM/PEI(1%)$_2$/GluOx$_5$/TEG(0.1%). Pt_c: Pt cylinder 1 mm long, 125 μm diameter; GluOx: L-glutamate oxidase; PPD:poly-ortho-phenylenediamine; polyZING: polymer from ZING; polyZINGDIM: polymer from ZING DIM; PEI: polyethyleneimine; TEG: triethyleneglycol. The subscript number indicates the number of dipping steps and in brackets, the concentration of the component. * $p < 0.05$ vs PPD; *** $p < 0.01$ vs PPD; **** $p < 0.001$ vs PPD.

Biosensor Design	V_{MAX} (nA)	K_M (μM)	LRS (nA/ μM)	LOD (μM)	LOQ (μM)	1 mM AA (nA)
**PPD-based**	192.6 ± 9.4	636.0 ± 81.3	0.164 ± 0.002	0.237 ± 0.001	0.791 ± 0.002	0.398 ± 0.163
**polyZING-based**	111.4 ± 5.1***	451.5 ± 41.2*	0.125 ± 0.003****	0.686 ± 0.004	2.285 ± 0.012****	5.307 ± 0.054****
**polyZINGDIM-based**	220.7 ± 9.0	783.7 ± 90.9	0.152 ± 0.004	0.701 ± 0.003	2.337 ± 0.009****	6.750 ± 0.687****
